# When normal ends at the median: a design flaw in child growth charts, India

**DOI:** 10.2471/BLT.26.295763

**Published:** 2026-08-20

**Authors:** Seetharaman Narayanan, Rajeshwari A Biradar, Yogeswaran Nehru Sivasubramanian

**Affiliations:** a Faculty of Public Health, Sri Balaji Vidyapeeth, Pillaiyarkuppam, , Pondicherry, 607 402, India.; b School of Public Health, Sri Balaji Vidyapeeth, Pondicherry, India.

## Abstract

India's Integrated Child Development Services programme monitors the growth of approximately 63 million children through a network of 1.4 million community-based childcare centres called Anganwadi Centres. The weight-for-age growth charts currently used in the programme contain an important design flaw in how normal growth is represented. While they incorporate the World Health Organization (WHO) standard-deviation (SD) cut-offs to identify levels of undernutrition (−2 SD and −3 SD), the green band denoting normal growth is visually truncated at the median (0 SD), thus omitting the upper half of the WHO-defined normal range (i.e. between the median and +2 SD). Our analysis of weight-for-age data of 209 043 children surveyed in India's National Family Health Survey-5 showed that 24 888 children (11.9%) fell above the median and below +2 SD. This design flaw would place an estimated 7.5 million children outside the programme chart's current normal growth band, despite them falling within the WHO-defined normal range. Official training materials offer no clear guidance on interpreting the growth of children that plot in this range. The same truncated growth chart appears in the Mother and Child Protection Card, used widely in India’s health-care system. This paper documents the design flaw, examines its implications, quantifies the scale of impact and makes the case for corrective action. The timing for addressing the flaw is opportune: Indian decision-makers are developing growth references for India under the *Upgrading Norms for Growth and Development Assessment of Indian Children *initiative.

## Résumé

Le programme indien des Services intégrés de développement de l’enfant (Integrated Child Development Services, ICDS) fait le suivi de la croissance d’environ 63 millions d’enfants grâce à un réseau de 1,4 million de centres communautaires de soins à l’enfance appelés « Centres Anganwadi ». Les courbes de croissance « poids pour l’âge » actuellement employées dans le cadre de ce programme présentent une faille de conception importante dans la manière dont la croissance normale est représentée. Bien qu’elles intègrent les seuils d’écart-type (ET) de l’Organisation mondiale de la Santé (OMS) pour identifier les niveaux de dénutrition (−2 ET et −3 ET), la bande verte indiquant une croissance normale est visuellement tronquée à la médiane (0 ET), omettant ainsi la moitié supérieure de la fourchette normale définie par l’OMS (c’est-à-dire entre la médiane et +2 ET). Notre analyse des données « poids pour l’âge » de 209 043 enfants interrogés dans le cadre de la 5^e^ Enquête indienne nationale sur la santé des familles a mis en évidence que 24 888 enfants (11,9%) se situaient au-dessus de la médiane et en dessous de +2 ET. Cette faille de conception placerait environ 7,5 millions d’enfants en dehors de la fourchette de croissance normale actuelle du tableau de croissance, alors même qu’ils se situent dans la fourchette normale définie par l’OMS. Les supports de formation officiels ne fournissent aucune indication claire sur l’interprétation de la croissance des enfants dont la courbe se situe dans cette fourchette. Ce même tableau de croissance tronqué figure sur la « Carte de protection de la mère et de l’enfant », largement utilisé dans le système de santé indien. Le présent article documente ce défaut de conception, se penche sur ses implications, quantifie l’ampleur de son impact et plaide en faveur de mesures correctives. Le moment est propice pour remédier à ce défaut: les décideurs indiens élaborent actuellement des références de croissance pour l’Inde dans le cadre de l’initiative « Mise à jour des normes d’évaluation de la croissance et du développement des enfants indiens ».

## Resumen

El programa de Servicios Integrados para el Desarrollo Infantil de la India supervisa el crecimiento de aproximadamente 63 millones de niños mediante una red de 1,4 millones de centros comunitarios de atención infantil denominados centros Anganwadi. Las gráficas de crecimiento de peso para la edad que se utilizan actualmente en el programa presentan un importante defecto de diseño en la representación del crecimiento normal. Aunque incorporan los puntos de corte de desviación estándar (DE) de la Organización Mundial de la Salud (OMS) para determinar los distintos grados de desnutrición (−2 DE y −3 DE), la franja verde que indica un crecimiento normal se trunca visualmente en la mediana (0 DE), por lo que omite la mitad superior del intervalo de normalidad definido por la OMS (es decir, entre la mediana y +2 DE). El análisis de los datos de peso para la edad de 209 043 niños incluidos en la quinta Encuesta Nacional de Salud Familiar de la India mostró que 24 888 niños (11,9%) se situaban por encima de la mediana y por debajo de +2 DE. Debido a este defecto de diseño, se estima que 7,5 millones de niños quedarían fuera de la franja de crecimiento normal de la gráfica utilizada actualmente por el programa, pese a encontrarse dentro del intervalo de normalidad definido por la OMS. Los materiales oficiales de formación no ofrecen orientaciones claras sobre cómo interpretar el crecimiento de los niños cuyos valores se sitúan en este intervalo. La misma gráfica de crecimiento truncada figura en la Tarjeta de Protección de la Madre y el Niño, ampliamente utilizada en el sistema sanitario de la India. En este artículo se documenta el defecto de diseño, se examinan sus implicaciones, se cuantifica la magnitud de su repercusión y se fundamenta la necesidad de adoptar medidas correctivas. Este es un momento oportuno para subsanar el defecto, ya que los responsables de la toma de decisiones de la India están elaborando referencias de crecimiento para el país en el marco de la iniciativa *Upgrading Norms for Growth and Development Assessment of Indian Children*.

## ملخص

يتولى برنامج الخدمات المتكاملة لتنمية الطفل في الهند مراقبة نمو ما يقرب من 63 مليون طفل من خلال شبكة تضم 1.4 مليون مركز لرعاية الأطفال على مستوى المجتمع المحلي، تُعرف باسم مراكز Anganwadi. إن مخططات نمو الوزن مقابل العمر المستخدمة حالياً في البرنامج تحتوي على خلل تصميمي دقيق يتعلق بكيفية تمثيل النمو الطبيعي. ففي حين تشمل هذه المخططات حدود الانحراف المعياري التي حددتها منظمة الصحة العالمية لتحديد مستويات نقص التغذية (عند -2 و-3 انحرافات معيارية)، فإن الشريط الأخضر الذي يشير إلى النمو الطبيعي يبدو مقطوعاً بصرياً عند القيمة الوسيطة (0 انحراف معياري)، مما يؤدي إلى استبعاد النصف العلوي من النطاق الطبيعي الذي حددته المنظمة (أي النطاق الواقع بين القيمة الوسيطة و+2 انحراف معياري). وقد أظهر تحليلنا لبيانات الوزن مقابل العمر الخاصة بـ 209043 طفلاً الذين شملهم المسح الوطني لصحة الأسرة في الهند (الجولة الخامسة)، أن 24888 طفلاً (أي %11.9) تقع قياساتهم فوق القيمة الوسيطة ودون مستوى +2 انحراف معياري. ومن شأن هذا الخلل التصميمي أن يضع ما يقدر بـ 7.5 مليون طفل خارج نطاق النمو الطبيعي المحدد حالياً في مخططات البرنامج، رغم أنهم يقعون فعلياً ضمن النطاق الطبيعي الذي حددته منظمة الصحة العالمية. ولا تقدم مواد التدريب الرسمية توجيهات واضحة بشأن تفسير نمو الأطفال الذين تقع قياساتهم ضمن هذا النطاق. ويظهر مخطط النمو المقطوع ذاته في بطاقة حماية الأم والطفل، المستخدمة على نطاق واسع في نظام الرعاية الصحية الهندي. وتوثق هذه الورقة البحثية الخلل التصميمي، وتناقش تداعياته، وتحدد حجم تأثيره، وتدعو إلى اتخاذ إجراءات تصحيحية. ويُعد التوقيت الحالي مناسباً جداً لمعالجة هذا الخلل، إذ يعمل صناع القرار في الهند حالياً على تطوير مراجع للنمو خاصة بالهند في إطار مبادرة تحديث معايير تقييم النمو والتطور لدى الأطفال الهنود.

## 摘要

印度的儿童发展综合性服务项目通过一个由 140 万个以社区为基础的儿童护理中心（被称作安甘瓦迪中心）组成的网络，来监测大约 6300 万名儿童的生长情况。这一项目目前使用的年龄别体重生长图表在如何呈现正常生长区间上包含一个重要的设计缺陷。虽然这些图表整合了世卫组织 (WHO) 标准差 (SD) 临界值来确定营养不良的等级（−2 标准差和 −3 标准差），但代表正常生长的绿色条带目测在中位数（0 标准差）处中断，因此略去了世卫组织 (WHO) 定义的正常范围的上半部分（即，中位数和 +2 标准差之间）。我们对印度第五次全国家庭健康调查中访问的 209043 名儿童的年龄别体重数据进行的分析显示，24888 名儿童 (11.9%) 处于中位数以上 +2 标准差以下。这一设计缺陷会将估计 750 万名儿童被置于该项目图表的当前正常生长条带以外，虽然他们应处于世卫组织定义的正常范围内。官方培训材料并未针对解读落在该区间内儿童的生长情况提供明确指导。同样中断的生长图表也出现于在印度医疗体系内广泛使用的母婴保护卡中。本文记录了这一设计缺陷、剖析其后果、量化其影响范围，并针对纠正措施进行论证。当下刚好是解决这一缺陷的合适时机：在印度儿童生长于发育评估规范升级倡议下，印度的决策者正在为印度制定生长参考值。

## Резюме

Индийская программа комплексного развития детей (Integrated Child Development Services) осуществляет мониторинг физического развития примерно 63 миллионов детей через сеть из 1,4 миллиона общественных центров по уходу за детьми, называемых центрами Анганвади. В настоящее время в рамках программы используются графики роста массы тела в зависимости от возраста, в которых содержится существенный недостаток в представлении нормы физического развития. Хотя на них используются пороговые значения стандартного отклонения (СО) Всемирной организации здравоохранения (ВОЗ) для определения степени недостаточности питания (−2 СО и −3 СО), зеленая полоса, обозначающая нормальное физическое развитие, визуально заканчивается на уровне медианы (0 СО), в результате из нее исключается верхняя половина диапазона нормы по определению ВОЗ (то есть значения между медианой и +2 СО). Анализ данных о массе тела в зависимости от возраста у 209 043 детей, обследованных в рамках пятого раунда Национального обследования здоровья семьи в Индии (NFHS-5), показал, что показатели 24 888 детей (11,9%) находились выше медианы и ниже +2 СО. Согласно оценкам, из-за этого конструктивного недостатка 7,5 миллиона детей окажутся за пределами текущей полосы нормального физического развития на графике программы, несмотря на то что их показатели находятся в пределах нормального диапазона, определенного ВОЗ. В официальных учебных материалах отсутствуют четкие указания относительно интерпретации показателей физического развития детей, попадающих в этот диапазон. Такой же график роста с усеченной полосой физического развития представлен в Карте охраны здоровья матери и ребенка (Mother and Child Protection Card), широко используемой в системе здравоохранения Индии. В данной статье описывается этот конструктивный недостаток, рассматриваются его последствия, оценивается масштаб его воздействия и обосновывается необходимость принятия корректирующих мер. В настоящее время сложились благоприятные условия для устранения этого недостатка: руководящие органы Индии разрабатывают национальные стандарты физического развития в рамках инициативы по актуализации норм оценки роста и развития индийских детей (Upgrading Norms for Growth and Development Assessment of Indian Children).

## Introduction

India’s Integrated Child Development Services programme, established in 1975, is one of the world’s largest platforms for monitoring early childhood development.^1^ Growth monitoring, using specific weight-for-age charts, is one of the cornerstones of the programme. Around 90 million beneficiaries are registered under the programme, of which about 63 million children younger than 5 years receive routine growth monitoring.^2^ Frontline workers conduct growth monitoring at community-based Anganwadi Centres. Children aged 3 years or younger are weighed monthly, while those aged 3 to 5 years are weighed once every 3 months; their weights are then plotted on growth charts.

The current weight-for-age growth charts used in the programme, which adopted the World Health Organization (WHO) child growth standards in 2009, use colour-coded bands to classify child growth: green for normal growth; yellow for moderate underweight; and orange for severe underweight.^3^ As well as allowing nutritional classification, the growth chart’s design also influences communication between the frontline workers and families. For example, if a child’s weight falls in the yellow or orange band, Anganwadi workers, who under the programme's recruitment rules are women, counsel and give appropriate guidance to families regarding the child’s growth status. For instance, they may give dietary advice if the child is moderately underweight; a child that is categorized as severely underweight or a child who is ill is referred to a primary health centre or nutrition rehabilitation centre, as set out in the programme’s growth monitoring and referral guidelines.^4^

## The design flaw

By definition, the median divides any distribution in half. According to WHO growth standards, normal weight range spans −2 standard deviation (SD) to +2 SD either side of the median, capturing approximately 95% of healthy, well-nourished children. Roughly 47.5% fall between −2 SD and the median and another 47.5% fall between the median and +2 SD.^5^

International growth charts generally represent the normal weight-for-age range as −2 SD to +2 SD (or equivalent percentiles).^6^^–^^11^ However, the weight-for-age charts the Integrated Child Development Services programme uses visually truncate the upper boundary of the green normal band at the median (0 SD). Consequently, children whose weight falls between the median and +2 SD are not represented within the chart’s designated normal band, despite being within the WHO-defined normal range.^3^ Programmatic guidance on the interpretation of measurements above the 0 SD line is unclear: official training materials do not clearly describe the distinction between children within the WHO-defined normal range (between 0 and +2 SD) and those above +2 SD.

Fig. 1 overlays the bands of the programme’s weight-for-age growth charts onto a standard normal distribution. Children whose weight-for-age falls between −2 SD and the median fall within the green area denoting normal. However, children falling between the median and +2 SD (shown in blue), who are statistically and biologically within the WHO-defined normal range, have no designated place on the current programme’s charts. Fig. 2 illustrates this missing representation through a side-by-side comparison of weight-for-age growth charts.

**Fig. 1:**
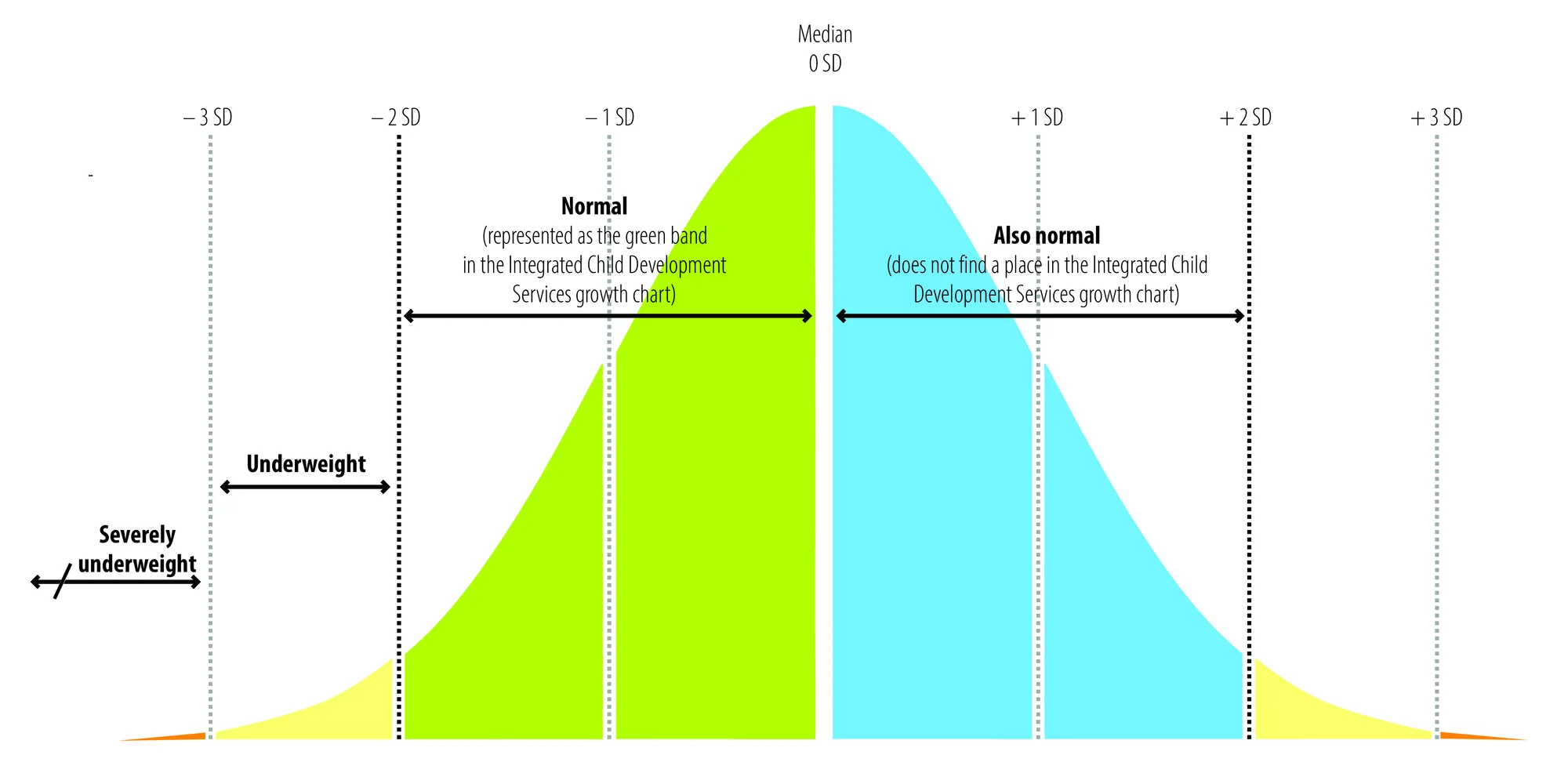
Integrated Child Development Services’ growth chart classification overlaid on a standard normal distribution, India

SD: standard deviation.

**Fig. 2:**
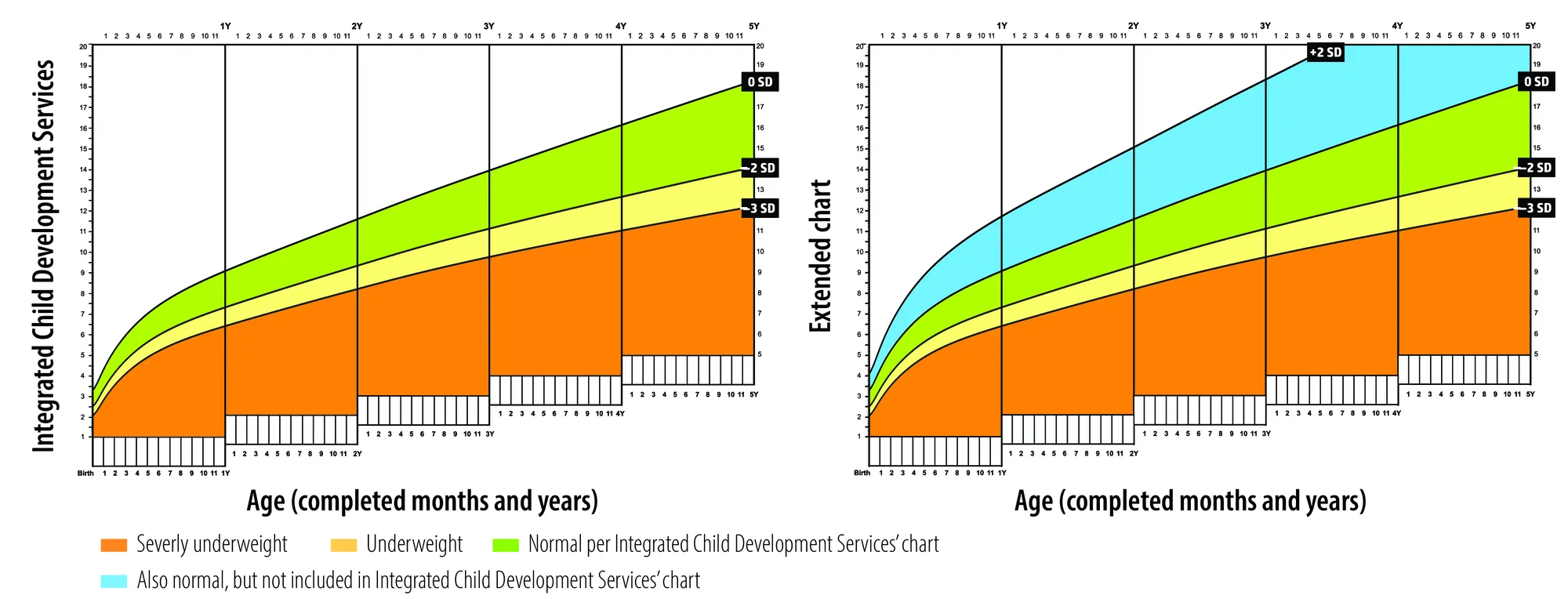
Comparison of India's current Integrated Child Development Services’ growth chart and a chart with the green band extended to +2 SD, India

SD: standard deviation.

This representation of normalcy in the programme’s growth charts differs from the approach used in several other countries that have also adapted WHO’s Child Growth Standards. A review that we conducted of growth charts from selected countries including Australia, Canada, Indonesia, Kenya, South Africa and United Kingdom of Great Britain and Northern Ireland demonstrates consistent inclusion of positive standard-deviation lines, thereby representing the full range of normal growth (i.e. from −2 SD through the median to +2 SD). Importantly, none of the charts reviewed treats the median as the upper limit of normal.

### Evolution of the growth charts

Before 2009, the Integrated Child Development Services programme used the Indian Academy of Pediatrics classification,^12^^–^^15^ based on the Harvard National Center for Health Statistics reference, to grade undernutrition. This classification system expressed weight-for-age as a percentage of the median, with the 50th percentile (median) curve as the upper reference and successive grades of underweight at roughly 80%, 70% and 60% of the median.^12^^–^^14^ Because the classification was built to grade undernutrition, its reference lines all sat at or below the median, which served as the upper reference; weights above it had no category on the chart. That classification was reasonable for a tool concerned only with growth failure, but it is where the truncation originates. When the programme adopted WHO’s Child Growth Standards in 2009, the transition was incomplete. While the lower boundaries in weight-for-age charts were updated to WHO's z-score framework (i.e. −2 SD and −3 SD), the upper boundary remained at the median rather than displaying the full, WHO-defined normal range.^5^^,^^15^^,^^16^ The transition resulted in a hybrid model that uses WHO's overall framework for growth monitoring but truncates the upper limit of normal growth in weight-for-age charts at the median.

### Accommodation in the guidelines

Two features of the official training materials show how the truncation is accommodated in routine use. First, the materials present the median as the upper limit of normal and, second, they improvise a workaround: an imagined hypothetical line above the green band for children whose weight plots above that limit.

#### Median as upper limit of normal

The programme’s growth monitoring and referral guidelines, published by the National Institute of Public Cooperation and Child Development, states:

“The 1st top curve line on the growth chart i.e. [the] upper border of [the] green band is the median which is, generally speaking, the average…”^4^

Having implied the median as the upper limit of normal (i.e. of the green band on the programme’s weight-for-age charts), the guidelines then proceed to explain what it means if a child’s weight falls above the green band:

“If [the] plotted weight of a child falls much above the 1st curve, the child has a growth problem, which can be overweight or obesity.”^4^

The guidelines do not clarify what “much above” means. Yet the chart's architecture leaves little ambiguity. With the median set as the upper boundary of the green band and no line above it marking the upper limit of the normal range, there is no zone in which weight falling above the median can be shown as normal. By default, this implies a growth problem.

#### The hypothetical line

Later sections of the guidelines, however, implicitly acknowledge that the median cannot serve as the upper limit of normal. Section 5.4 of the guidelines includes the following rather unclear passage:

“[If a] child’s growth curve is far above the first curve line: [the] child may have a growth problem… However, it may be mentioned that a tentative principle has been followed to understand the growth curve above the green band that is a hypothetical line dividing [the] green band into two equal parts may be drawn and one of the two equal parts may be pasted above the green band and the children whose plotting falls between this hypothetical line and the real green zone [band] need not to be referred to [a] health centre. However, children whose plotting falls above the hypothetical line actually have [a] growth problem and need to be referred to [a] health centre.”^4^

In effect, the guidelines ask Anganwadi workers to first imagine a line dividing the green band in half and then to project a band half the width of that above the chart's current green boundary. The worker then needs to judge whether a child's plot falls within or above this imagined zone. They are then expected to base their referral decisions on this judgement.

The workaround is itself evidence of the design flaw. In devising it, the guidelines effectively concede that the median cannot be the upper limit of normal, yet instead of printing the +2 SD line that marks where the normal range actually ends, they ask frontline workers to reconstruct a boundary in their heads: halving the green band and projecting that half above the median. This workaround is cumbersome and an unrealistic expectation of a frontline worker. The workaround also places the action threshold at an improvised boundary above the median, rather than at a specified weight-for-age z-score. Thus the system recognizes the problem without resolving it, substituting an imagined and misplaced boundary for the printed +2 SD line that would settle the matter. The wording for the workaround also appears, verbatim, in the National Institute of Public Cooperation and Child Development's frequently asked questions on growth monitoring.^17^ Repetition of this passage across two separate official documents marks it as official guidance rather than an isolated inconsistency.

### A lack of debate

The most striking aspect about the design flaw is not what has been written, but what has not been written, during the more than15 years since the adoption of the revised growth charts. Searches of published literature, government reports and of publicly available policy documents identified no published critique, technical memorandum or policy brief regarding the truncated normal range or the deviation from WHO standards. We also found no documentation defending this design choice. Even the most comprehensive government-commissioned evaluation of the Integrated Child Development Services programme does not address growth chart architecture.^1^ This silence is neither documented disagreement nor reasoned controversy; we found no published discussion of the truncation or its implications.

### Explanations and counterarguments

In the absence of published literature, we were informally offered explanations by paediatricians, public health academics and personnel involved in programme implementation (Box 1). These explanations were not collected through any formal study; they were offered informally in academic discussions and in response to presentations among colleagues. None of the explanations appear in any official document, they emerged only after we highlighted the problem, suggesting that they are post hoc rationalizations rather than deliberated design rationale. We should emphasize that even if the programmatic focus of the Integrated Child Development Services programme remains on undernutrition, growth charts function as normative tools. Design choices, particularly the truncation of the normal band at the median, shape how normal growth is defined and interpreted in practice. Capping normal at the median in effect lowers the threshold at which a child is treated as having a growth problem; lowered or shifted diagnostic thresholds are a recognized driver of overdiagnosis and its downstream harms.^18^ Note that our study does not advocate obesity screening using weight-for-age (which is an inappropriate indicator for that purpose). Rather, it advocates for accurate depiction of the full range of normal growth.

Box 1 **Explanations offered for the chart design and our counterarguments**

***“Integrated Child Development Services primarily targets underweight detection, so the upper boundary doesn't matter.”***

We counterargue that growth monitoring requires knowing what is normal, not only what constitutes growth failure. Anganwadi workers need to reassure families when growth is normal.

**“*Overweight is a minor concern in India.”***

We argue that children whose weight-for-age falls in the range between 0 SD and +2 SD are within the normal weight-for-age range but are placed outside the chart's normal band. Weight-for-age alone cannot determine overweight.

***“Additional lines would overwhelm frontline workers.”***

We argue that the system already asks Anganwadi workers to imagine the lines. The proposed change removes the need to imagine a boundary above the median and recommends printing the +2 SD line, rightfully extending the upper limit of normal growth. This is simpler than the present workaround. Also note that Indonesia, Kenya and South Africa use complete charts with comparable workforces.^9^^–^^11^

***“The Indian Council of Medical Research's forthcoming charts will anyway resolve this.”***

India’s own reference charts taking shape under the Upgrading Norms for Growth and Development Assessment of Indian Children programme are unlikely before 2028–2029; waiting for these means several more years during which millions of children a year will continue to be classified on a chart that omits half the normal range. Besides, if we do not recognize and fix the flaw being reported, there is no guarantee that the new charts will avoid the same error.

***“A flaw this fundamental could not have persisted unnoticed; those who designed and approved the charts must have had sound reasons.”***

This comment is a presumption of prior diligence, not a documented rationale. We found no record of the upper boundary of normal growth being questioned, much less examined and resolved. The belief that an established tool must already have been scrutinized (and that questioning it falls outside one's standing) partly explains how the truncation has endured unexamined.

SD: standard deviation.Note: explanations informally offered by paediatricians, public health academics and personnel involved in programme implementation.

## Implications of the truncation

The design flaw is not merely presentational: the programme’s growth chart provides no normal zone in which to place a child who plots above the median yet within the WHO-defined normal range. Such a child gives the Anganwadi worker three unsatisfactory options. She may, uncertain how to read the plot, treat the weight as falling outside the normal range and refer the family to a health centre, adding to the demands on an already strained primary care system. She may, acting on the same misreading, give the family unnecessary counselling or cautionary messages about a child who is, by accepted WHO standards, growing normally. Or she may, consciously or otherwise, adjust her own recording to avoid the recurrent discrepancy, plotting selectively, rounding weights down, or attending less closely to the chart. We did not assess these practices directly, but they are consistent with existing evidence.^19^^–^^22^ Each of these responses undermines the purpose of systematic growth monitoring, and each is sensitive to where the threshold for concern is set. That sensitivity is considerable: in one study, applying different growth-screening criteria to the same children shifted the false-positive referral rate from under 1% to nearly 80%.^19^ The burden is not hypothetical, Anganwadi workers already report heavy administrative workloads,^20^^–^^22^ and further ambiguity in chart interpretation compounds this. Nor is the cost only operational: when families of visibly healthy children are warned or referred without cause, confidence in growth monitoring erodes.

The truncation is not confined to the programme’s chart. The same median-capped weight-for-age chart is printed in the Mother and Child Protection Card,^16^ a family-held record issued jointly for the programme and the primary health-care system.^23^ This replication means that the truncated depiction reaches the health system and families, as well as the Anganwadi network. The flawed definition of normal is therefore embedded in the routine growth-monitoring instruments of both of India's principal maternal and child health platforms.

## The scale of impact

Although the truncation may appear a minor design choice, it shapes how frontline workers at 1.4 million Anganwadi Centres interpret and respond to children's growth. To quantify how many children are potentially affected by the design flaw, we analysed anthropometric data from India’s National Family Health Survey-5, a nationally representative survey conducted from 2019 to 2021.^24^ This analysis quantifies the scale of potential misclassification; it does not directly measure downstream effects such as referrals, counselling practices or family concerns.

For our analysis we extracted weight-for-age data for 209 043 children aged 0–59 months (107 846 male; 101 197 female) after excluding biologically implausible values using WHO flagging criteria (i.e. weight-for-age z-scores below −6 SD or above +5 SD).^25^ Table 1 presents the distribution by z-score category. The 24 888 children (11.9%) with weight-for-age values falling between 0 SD and +2 SD represent the population potentially affected by the growth chart design flaw. By WHO standards, these children fall within the normal weight-for-age range; on the current programme’s charts their measurements plot outside the band designated as normal. Fig 3 shows weight-for-age data of 101 197 girls from India's National Family Health Survey-5 plotted onto the current programme’s growth curves. The entire blue band represents children who plot above the programme chart's normal band.

**Table 1:** Distribution of children's weight-for-age z-scores, National Family Health Survey-5, India, 2019–2021

Category	z-score range	No. of children (%) (*n* = 209 043)
**Severely underweight**	< −3 SD	20 254 (9.69)
**Underweight^a^**	< −2 SD	63 562 (30.41)
**Normal as per WHO**	−2 SD to +2 SD	142 943 (68.38)
Normal as per the programme	−2 SD to 0 SD	118 055 (56.47)
Normal missed by the programme	0 SD to +2 SD	24 888 (11.91)
**Above +2 SD**	> +2 SD	2 538 (1.21)

SD: standard deviation; WHO: World Health Organization.

^a^ Includes children who are severely underweight (< −3 SD).

Note: the programme refers to India’s Integrated Child Development Services programme.

**Fig. 3:**
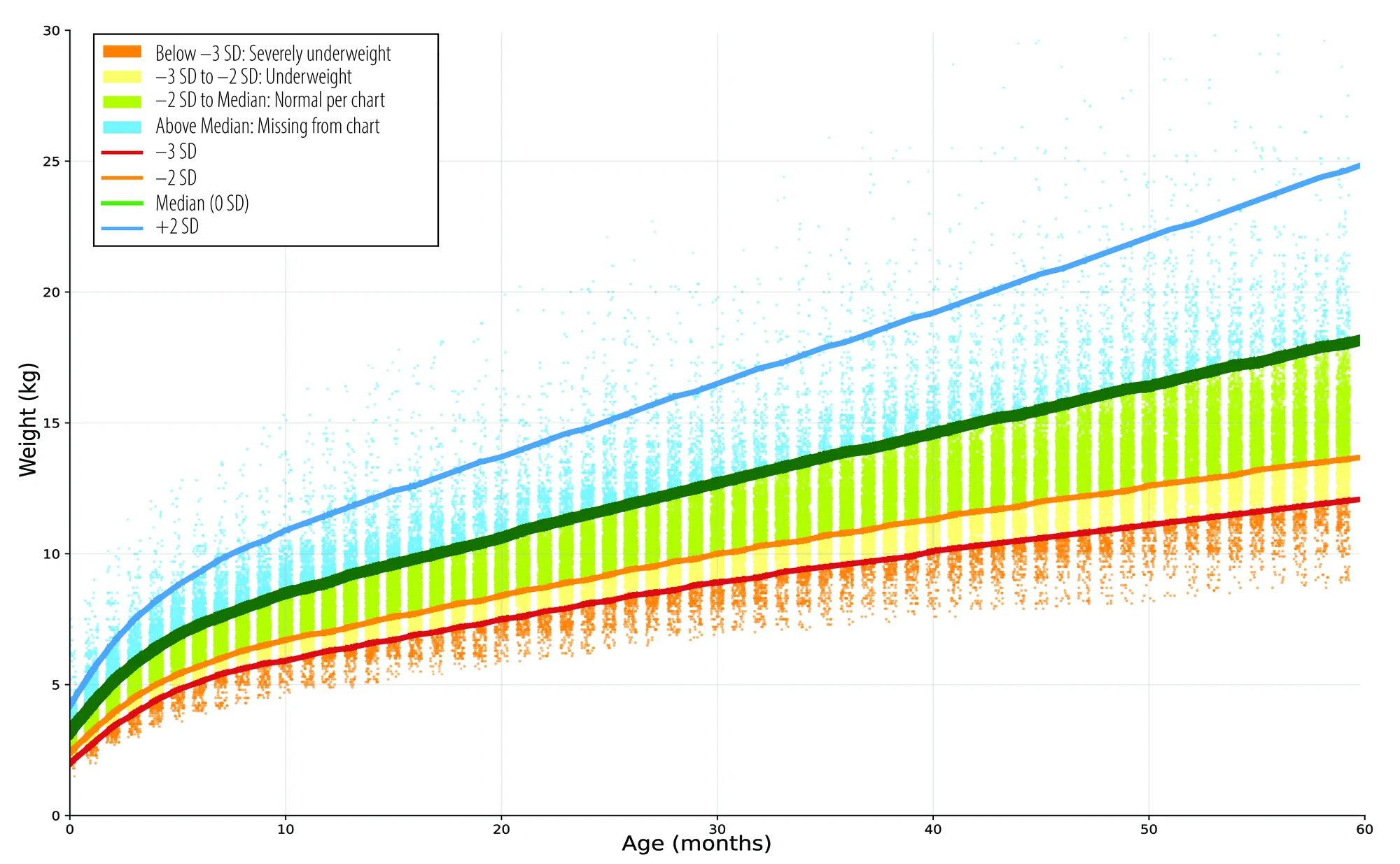
Girls' weight-for-age plotted against India's Integrated Child Development Services growth chart, India, 2019–2021

SD: standard deviation.

Notes: the survey collected information on 101 197 girls aged 0–59 months. Blue points represent all observations above the median, including those above +2 SD. The subgroup between the median and +2 SD is the focus of the present analysis.

Data source: National Family Health Survey-5.^24^

### Potential impact nationally

The programme conducts growth monitoring of approximately 63 million children younger than 5 years. Applying the 11.9% proportion derived from our analysis of survey data to this figure (0.119 × 63 million), we estimate that approximately 7.5 million children nationally would fall outside the chart's designated normal band. This estimate should be read as an order-of-magnitude estimate rather than a precise count as the survey sample may differ in socioeconomic and nutritional profile from the programme’s beneficiary population. These children are weighed regularly, their growth plotted, and their families counselled on the basis of charts that recognize only half of the normal range, all because of a missing chart line.

## Corrective action

The most immediate corrective step is a single change to the existing charts: extend the green band to +2 SD, so that the upper boundary of normal is printed where the WHO standards place it. The median line remains on the chart as a reference for interpreting trend; it ceases to function as a boundary. Because the same chart appears in the Mother and Child Protection Card, the correction should extend there too: revising the chart alone would leave the truncated depiction in the family-held record and in the copies used across the health system.^16^ The printing modification should be feasible within routine chart revision and printing processes. Implementing and normalizing this change would, however, require focused in-service retraining and supportive supervision to ensure that Anganwadi workers interpret plots falling above the median and up to +2 SD as normal.

We do not recommend formalizing the improvised boundary described in the current guidance. Printing that boundary while retaining the instruction to refer children plotting above it would risk perpetuating an inadequately specified referral threshold. 

### Time to act

The persistence of the truncated normalcy for more than 15 years may reflect the complexity of growth monitoring within large-scale programmes, where chart design, training, supervision and service delivery operate across multiple levels of implementation.

The Indian Council of Medical Research has embarked on the UNNATI initiative (Upgrading Norms for Growth and Development Assessment of Indian Children) to develop country-specific growth references for Indian children.^26^^,^^27^ As this framework is unlikely to be implemented before 2028–2029, there is an opportunity to ensure that the design flaw identified here is not carried forward into India’s new growth monitoring framework. But there is no need to wait for the initiative to act on the identified flaw: existing charts can readily be corrected at the next routine reprint by extending the green band to +2 SD and revising the accompanying training materials.

Note that discussions about the choice of country-specific growth references vis-a-vis universal growth standards like WHO’s^28^ are legitimate scientific and policy questions beyond the scope of this paper. Our concern is more foundational: whatever system India adopts, the resultant growth charts must depict the complete range of normalcy.

## Conclusion

For more than 15 years, the Integrated Child Development Services programme has plotted children's growth on charts where the green normal band stops at the median. This design flaw places millions of children with normal weight-for-age outside the chart's designated normal band. Because the same chart is in the Mother and Child Protection Card, this incomplete representation extends beyond the programme to the health system and to families themselves.

The solution is straightforward: extend the green band of normal growth to +2 SD on the existing charts, revise the training materials to match, and ensure the UNNATI initiative depicts the full range of normal growth from the outset. We offer our analysis in the spirit of strengthening India's nutrition surveillance infrastructure, rather than faulting it. Moving one boundary is a small correction with a wide reach, and a chance to ensure the next generation of growth charts is complete from the start.
